# Automated left heart chamber volumetric assessment using three-dimensional echocardiography in Chinese adolescents

**DOI:** 10.1530/ERP-17-0028

**Published:** 2017-09-01

**Authors:** Xiu-Xia Luo, Fang Fang, Hung-Kwan So, Chao Liu, Man-Ching Yam, Alex Pui-Wai Lee

**Affiliations:** 1 Department of Ultrasonography, Shenzhen Hospital, Southern Medical University, Shenzhen, People’s Republic of China; 2 Division of Cardiology, Department of Medicine and Therapeutics, Prince of Wales Hospital, Faculty of Medicine, The Chinese University of Hong Kong, Hong Kong, People’s Republic of China; 3 Beijing Institute of Heart, Lung and Blood Vessel Diseases, Beijing Anzhen Hospital, Capital Medical University, Beijing, People’s Republic of China; 4 Department of Pediatrics, Prince of Wales Hospital, The Chinese University of Hong Kong, Hong Kong, People’s Republic of China

**Keywords:** three-dimensional echocardiography, HeartModel, automation, cardiac chamber quantification, adolescents

## Abstract

**Background:**

Several studies have reported the accuracy and reproducibility of HeartModel for automated determination of three-dimensional echocardiography (3DE)-derived left heart volumes and left ventricular (LV) ejection fraction (LVEF) in adult patients. However, it remains unclear whether this automated adaptive analytics algorithm, derived from a ‘training’ population, can encompass adequate echo images in Chinese adolescents.

**Objectives:**

The aim of our study was to explore the accuracy of HeartModel in adolescents compared with expert manual three-dimensional (3D) echocardiography.

**Methods:**

Fifty-three Chinese adolescent subjects with or without heart disease underwent 3D echocardiographic imaging with an EPIQ system (Philips). 3D cardiac volumes and LVEF obtained with the automated HeartModel program were compared with manual 3D echocardiographic measurements by an experienced echocardiographer.

**Results:**

There was strong correlation between HeartModel and expert manual 3DE measurements (*r* = 0.875–0.965, all *P* < 0.001). Automated LV and left atrial (LA) volumes were slightly overestimated when compared to expert manual measurements, while LVEF showed no significant differences from the manual method. Importantly, the intra- and inter-observer variability of automated 3D echocardiographic model was relatively low (<1%), surpassing the manual approach (3.5–17.4%), yet requiring significantly less analyzing time (20 ± 7 vs 177 ± 30 s, *P* < 0.001).

**Conclusion:**

Simultaneous quantification of left heart volumes and LVEF with the automated HeartModel program is rapid, accurate and reproducible in Chinese adolescent cohort. Therefore, it has a potential to bring 3D echocardiographic assessment of left heart chamber volumes and function into busy pediatric practice.

## Introduction

Precise and fast as well as reproducible measurements of left atrial (LA) and left ventricular (LV) volumes and systolic function are the most common and important tasks of transthoracic echocardiography (TTE), which is clinical relevant in the diagnosis, prognosis and risk stratification of various congenital and acquired heart diseases in the young. Conventional M-mode and two-dimensional echocardiography (2DE) are the most ubiquitous and non-invasive tools for chamber quantification and cardiac function, the aforementioned methods however are limited by geometric assumption, image foreshortening and inter/intra-observer variability, and thus compromise the accuracy and reproducibility in clinical decision making and research investigations (1, 2, 3). Recently, full-volume three-dimensional (3D) echocardiography (3DE) has been introduced to quantify LA or LV volumes, which overcomes disadvantages of conventional echocardiography. Previous studies have demonstrated the superiority in accuracy and reproducibility of 3DE over 2DE for the assessments of left heart volumes and left ventricular ejection fraction (LVEF) (4, 5, 6, 7, 8). However, widespread application of 3DE in clinical practice is hampered by the time-consuming workflow and need for 3DE-specific expertise (9, 10, 11, 12).

Consequently, with evolvement in Anatomical Intelligence Ultrasound (AIUS), a novel automated 3DE software (HeartModel) emerged and offers an option of simultaneous quantification of LA and LV volumes and LVEF within seconds avoiding any human interaction. HeartModel is reliable to measure LA and LV volumes in adults (age >35 years), which has been validated with cardiac magnetic resonance (CMR) (13, 14, 15). Nevertheless, no data are available in the Chinese adolescents with or without congenital heart disease. Of note, this prototype program uses a unique adaptive analytics algorithm that relies on an affluent 3DE database with a wide range of morphologies derived from a ‘training’ population, which may not adequately encompass the Chinese adolescent cohort, who usually have smaller cardiac chamber size than their Western counterparts (16). Moreover, the smaller heart chamber size may increase the difficulty in delineating the endocardial border in the youth.

Thus, the aim of this study was to explore the accuracy and reproducibility of the HeartModel program for automated measurement of LA, LV volumes and LVEF from 3DE datasets in the adolescents, using expert manual 3DE as reference.

## Methods

### Study population

This study was a part of a community-based follow-up study for persistent masked hypertension in Hong Kong pediatric population in 2015/2016. The inclusion criteria were adolescents with persistent masked hypertension who had been initially screened from the Hong Kong community in 2011/2012 according to the local ambulatory blood pressure reference (17). The community controls were also recruited. Therefore, from November 2015 to July 2016, a total of 58 consecutive young subjects (age ranging from 13 to 22 years) with comprehensive 3DE images were selected, including 12 patients with persistent masked hypertension, 2 patients with congenital heart disease (secundum atrial septal defect and mild pulmonary stenosis) and 44 normal subjects. After excluding five subjects with poor image quality, 53 individuals were studied, including 19 with excellent images (36%), 28 with good images (53%) and 6 with fair images (11%). The exclusion criterion was the presence of a poor acoustic window or patient unwillingness. This study protocol was approved by the Joint the Chinese University of Hong Kong and New Territories East Cluster Clinical Research Ethics Committee and the Ethics Committee of the Department of Health of the Hong Kong Government (CRE-2013.563). Written informed consent was obtained from the parents of participants aged below 18 years.

### Echocardiographic image acquisition

Comprehensive transthoracic with standard 3D echocardiography was performed using an EPIQ ultrasound system with X5-1 matrix array transducer (Philips Healthcare). For 3DE section, imaging settings were optimized for endocardial visualization before each acquisition. A novel single-beat acquisition mode (HM ACQ key on EPIQ 7C) was utilized to obtain one-beat full-volume 3DE datasets. Special care was taken to include the entire LA and LV cavity within the pyramidal 3D volume. At least three dynamic pyramidal datasets were acquired and stored for each individual, and the best single-beat 3DE full-volume dataset was selected for offline analysis. All 3D images were respectively analyzed offline with both manual (3DQA, QLAB 10.5; Philips Healthcare) and prototype-automated software packages (HeartModel, QLAB 10.5; Philips Healthcare) by an experienced reader to calculate LV/LA volumes and LVEF.

### Automated 3DE measurements

**Figure 1:**
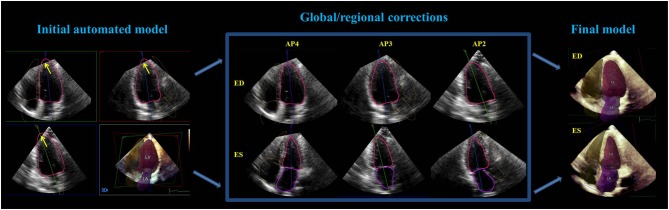
A case showed how the manual corrections were performed to improve the automated endocardial tracing. Yellow arrows showed the region that required contour editing after initial fully automated detection of left ventricular (LV) and left atrial (LA) endocardial surfaces (left). The optional corrections were respectively done in the non-foreshortened 2D cut-planes that automatically extracted from the 3D datasets at end-diastole (center, top) and end-systole (center, bottom), and then the final 3D shell of the cardiac chambers were reconstructed (right). #, number of chambers; AP, apical view; ED, end-systole; ES, end-systole.

Using an adaptive analytics algorithm, the HeartModel program automatically determines global cardiac shape orientation and detects LA and LV endocardial surfaces from a 3DE database, containing approximately 1000 3D-TTE datasets of varying image quality in patients with a wide range of function and morphologies. The program matches relevant image features of the given LV volume to the database. This selected model is then locally adapted to the LV volume under study using a series of adaptations. LV end-diastolic volume (EDV) was selected using motion analysis near the peak of the electrocardiographic R-wave. LV end-systolic volume (ESV) was determined using motion analysis to identify the minimal volume (9, 14). The endocardial boundary recognition was set as 40% at end-diastole (ED) and 8% at end-systole (ES) in advance by our center, and then applied in all automatic analysis process. Although the ED and ES frames and endocardial contours could be modified, only three manual corrections were made for our primary analysis. Finally, a static 3D shell of the LV and LA cavity was reconstructed and parameters including LVEDV, LVESV, LVEF and LA volume (LAV) at LVES were automatically calculated (Fig. 1). The time required to complete the automatic volumetric analysis from 3DE datasets was recorded.

### Manual 3DE measurements

Manual 3D quantification of LV volumes and LVEF as well as LAV at LVES was performed offline on a QLAB workstation (3DQA, QLAB 10.5; Philips Healthcare) by an experienced investigator blinded to the results of automated 3D model. Cardiac chamber quantification starts by aligning the extracted LV 4-chamber and orthogonal views to avoid foreshortening. Subsequently, the end-diastolic (largest LV volume) and end-systolic (smallest LV volume) frames are identified. On both ED and ES frames, 4 mitral annular and 1 apical points were then placed on the LV as landmarks in each of the views. LV endocardial contours were tracked in every slice semi-automatically frame by frame throughout the entire cardiac cycle. Unsatisfactory delineation of the endocardial border was manually edited, and the final LVEDV, LVESV and LVEF were then displayed. For the manual 3D measurement of LA volume, the long-axis of the LA was identified at LVES in the 4- and 2-chamber cut-planes, and the blood–tissue interface was traced semi-automatically to obtain maximum LAV. The time required to manual measurements of LVEDV, LVESV, LVEF and LAV at LVES from 3DE datasets was also recorded.

### Reproducibility analysis

To determine the reproducibility of 3D volumetric quantification by each imaging modality, HeartModel and manual 3DE analysis were repeated in a randomly selected group of 30 study subjects by another investigator (novice) as well as by the same primary reader (expert) at least 10 days later. Inter-observer and intra-observer variability were calculated as the mean percentage error, defined as the absolute difference of the corresponding pair of repeated measurements as a percentage of their mean in each patient and then averaged over the study group.

In addition, test–retest reproducibility was assessed in 30 separate subjects. After obtaining the initial 3DE dataset, the sonographer stopped echo scanning for 5 min, and then repositioned the subject and the transducer to obtain a second dataset. LV/LA volumes and LVEF were measured in a fully automated manner as described here. During all repeated analyses, the investigators were blinded to the results of all previous measurements.

### Statistical analysis

Statistical analysis was performed with the IBM SPSS Statistics, version 21 (IBM) software. Continuous variables are expressed as mean ± s.d. and nominal variables as percentages. The agreement of HeartModel program with the expert manual 3DE reference values for LVEDV, LVESV, LVEF and LAV was evaluated using Bland–Altman analysis to assess the bias (mean difference) and the limits of agreement (LOA, 1.96 s.d.s around the mean difference). To verify the significance of the biases, paired *t*-test with a two-tailed distribution was performed. The relationship between HeartModel and the manual measurements was evaluated using linear regression with Pearson correlation coefficients. The paired *t*-test was also used to compare chamber volumes and LVEF between the two analyzing programs. Two-tailed *P* values <0.05 were considered statistically significant.

## Results

Patient characteristics are presented in Table 1. 3D cardiac quantification by HeartModel software was feasible in 53 out of 58 (91%) subjects (17 ± 3 years, 31 males). Five patients were excluded due to poor image quality. Automated analysis with contour adjustment was only performed in three subjects (6%). The average 3DE frame rate was 21 ± 1 Hz (ranged from 18 to 23 Hz).

**Table 1:** Clinical and demographic characteristics of the study population.

**Parameters**	
Ages (years)	17 ± 3
Male, *n* (%)	31 (58)
Body surface area (m^2^)	1.61 ± 0.21
Body mass index (kg/m^2^)	21 ± 3
Heart rate (bpm)	77 ± 11
Systolic blood pressure (mmHg)	112 ± 10
Diastolic blood pressure (mmHg)	64 ± 8
Left ventricular ejection fraction (%)	62 ± 4

Data expressed as mean ± s.d. or number (percentage).

### HeartModel vs expert manual 3DE measurements

**Figure 2:**
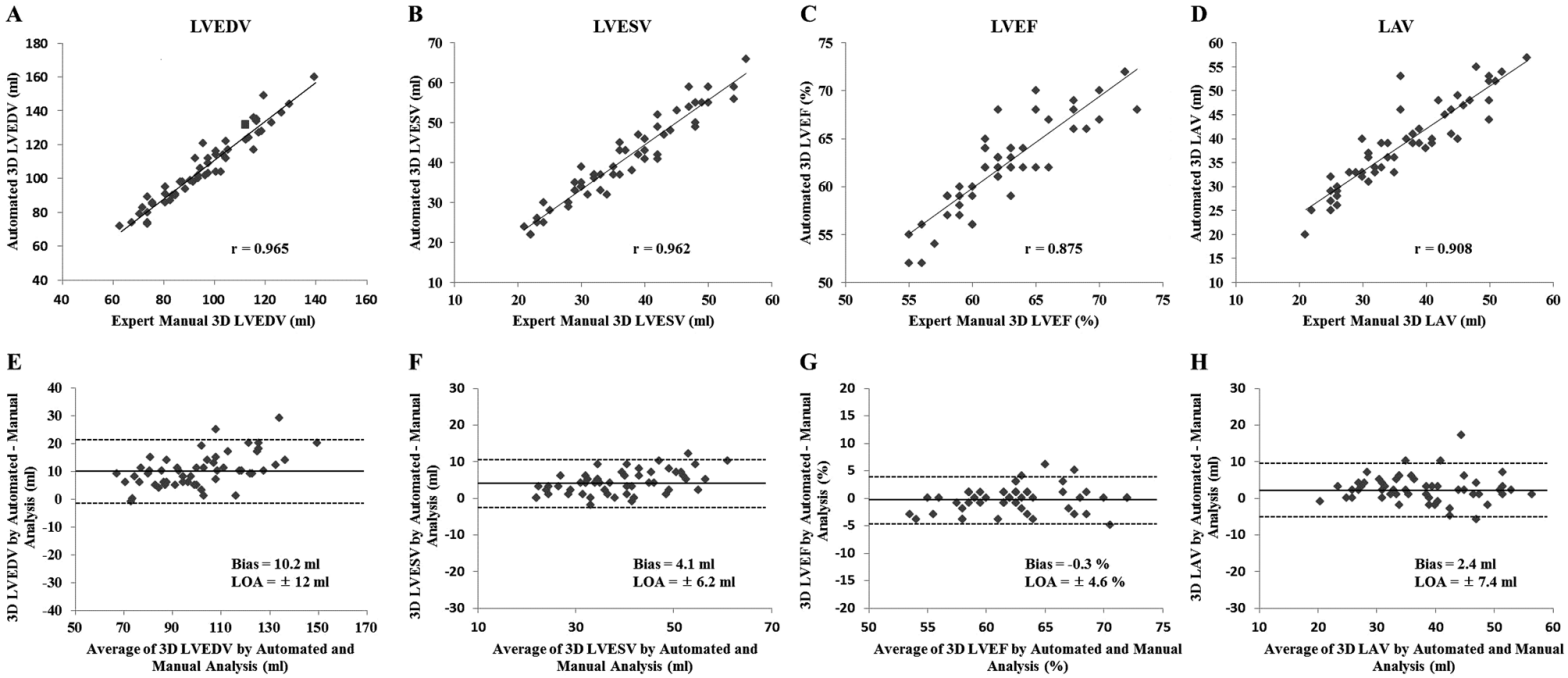
Correlation (top) and Bland–Altman analysis (bottom) of comparison between automated HeartModel program and expert manual 3D method for (A, E) left ventricular end-diastolic volume (LVEDV), (B, F) left ventricular end-systolic volume (LVESV), (C, G) left ventricular ejection fraction (LVEF), and (D, H) left atrial volume (LAV) at left ventricular end-systole (LVES). Solid lines indicate bias and dashed lines indicate limits of agreement (LOA).

3DE measurements assessed by HeartModel and expert manual method are depicted in Table 2. Overall, there was a good correlation between HeartModel and expert manual 3DE measurements for estimation of LVEDV, LVESV, LVEF and LAV at LVES in all studies subjects (*r* = 0.875–0.965, all *P* < 0.001) (Fig. 2A, B, C and D). The HeartModel-derived LV volumes and LA volume were slightly larger than the expert manual 3DE measurements (all *P* < 0.001) (Table 2). However, LVEF determined by the automated 3DE model was similar to the manual values (*P* = 0.38) (Fig. 2G and Table 2). Mean difference as performed with Bland–Altman was 10.2 ± 6.1 mL (LOA: −1.8 to 22.3 mL) for LVEDV, 4.1 ± 3.2 mL (LOA: −2.1 to 10.3 mL) for LVESV, −0.3 ± 2.3% (LOA: −4.9 to 4.3%) for LVEF and 2.4 ± 3.8 mL (LOA: −5.0 to 9.9 mL) for LAV at LVES (Fig. 2E, F, G and H).

**Table 2:** HeartModel comparison vs expert manual 3DE measurements.

	**HeartModel**	**Expert manual 3DE method**	***P***	**Bias**	**LOA** (1.96 s.d.s)	**Correlation**
LVEDV (mL)	106.9 ± 21.2	96.6 ± 17.9	<0.001	10.2	12.0	0.965
LVESV (mL)	40.9 ± 10.8	36.7 ± 9.3	<0.001	4.1	6.2	0.962
LVEF (%)	62.2 ± 4.8	62.4 ± 4.4	0.381	−0.3	4.6	0.875
LAV at LVES (mL)	38.9 ± 8.8	36.5 ± 9.0	<0.001	2.4	7.4	0.908

* Paired *t*-test comparing HeartModel and expert manual measurements.

3DE, three-dimensional echocardiography; LAV, left atrial volume; LOA, limits of agreement; LVEDV, left ventricular end-diastolic volume; LVEF, left ventricular ejection fraction; LVES, left ventricular end-systole; LVESV, left ventricular end-systolic volume.

### Reproducibility

The results of the reproducibility analysis were summarized in Table 3. Even performed by two observers in different levels of experience, the reproducibility of cardiac volumes and LVEF with automated 3DE model even with minor contour adjustment was excellent, as the intra- (0.07–0.72%) or inter-observer (0–0.48%) variability was minimal. On the other hand, both intra-observer (3.5–8.3%) and inter-observer (10.5–17.4%) variability for manual 3DE measurements were significantly higher than the automated values.

**Table 3:** Reproducibility.

	**HeartModel**			**Manual 3DE method**	
**Variable**	Intra-observer	Inter-observer	Test–retest	Intra-observer	Inter-observer
LVEDV (%)	0.15 ± 0.57	0.16 ± 0.86	3.4 ± 3.7	4.9 ± 3.6	10.5 ± 6.9
LVESV (%)	0.72 ± 2.92	0.48 ± 2.61	5.1 ± 5.1	8.3 ± 5.9	13.1 ± 11.4
LVEF (%)	0.69 ± 2.82	0.20 ± 1.09	2.5 ± 2.1	3.5 ± 3.2	10.8 ± 9.3
LAV at LVES (%)	0.07 ± 0.40	0 ± 0	5.9 ± 6.3	4.8 ± 5.0	17.4 ± 20.1

Values are mean ± s.d.

Abbreviations as in Table 2.

**Figure 3:**
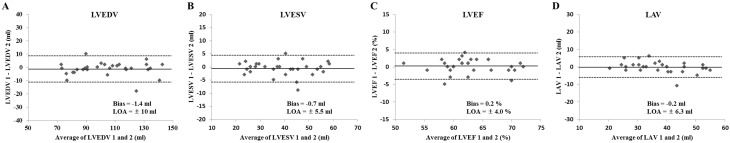
Test–retest reproducibility of HeartModel program, using Bland–Altman plot for LVEDV, LVESV, LVEF and LAV at LVES in 30 adolescent subjects. Abbreviations as in Fig. 2.

Test–retest variability (2.5–5.9%) of the automated program by using a different dataset was higher than the inter-observer variability. The LVEDV, ESV, LVEF and LAV at LVES for the first and second measurements were as follows: 104 ± 22 mL vs 105 ± 22 mL (*P* = 0.14), 39 ± 11 mL vs 40 ± 11 mL (*P* = 0.20), 63 ± 5% vs 63 ± 5% (*P* = 0.66), and 38 ± 9 mL vs 38 ± 10 mL (*P* = 0.69). The corresponding Pearson *r* values were 0.97, 0.97, 0.92 and 0.95 (all *P* < 0.001), respectively. Bland–Altman analysis showed minimal bias between the two measurements with excellent agreement (Fig. 3).

### Examination duration

**Figure 4:**
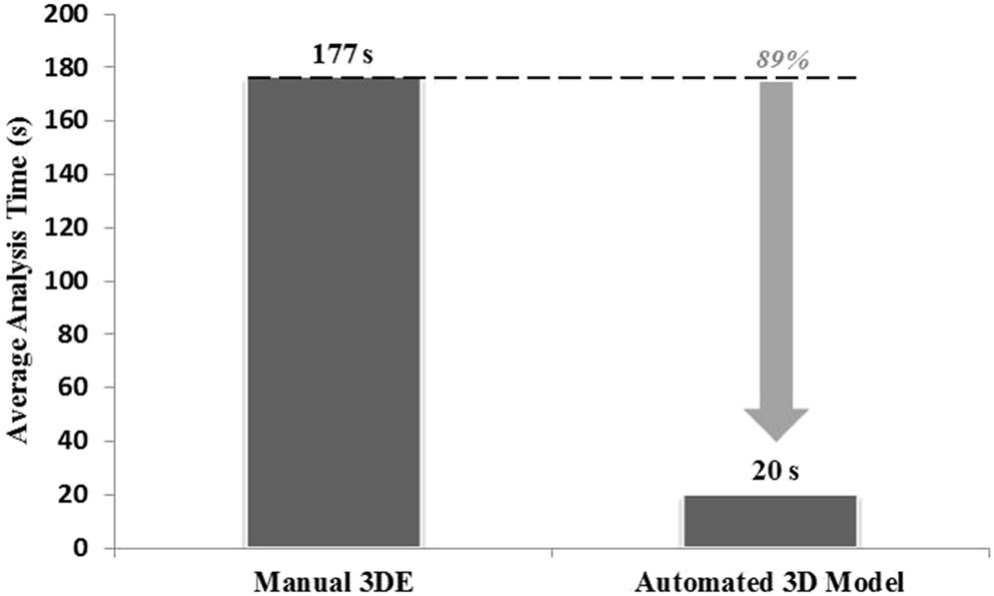
3DE volumetric analysis time of left heart chambers. With manual 3DE analysis as the actual reference, a decrease in analysis time of 89% for automated HeartModel program was noted.

The average time needed for volumetric analysis was significantly shorter using HeartModel compared to manual 3DE method (20 ± 7 vs 177 ± 30 s, *P* < 0.001), approximately a tenth of the time using manual method (Fig. 4). If HeartModel program was carried out without contour adjustment, the analysis time was reduced even more (19 ± 2 s, *P* < 0.001).

## Discussion

This is the first study to investigate the utility of the automated HeartModel program in simultaneous quantification of cardiac volumes and function in the adolescent cohort.

### Automated LV analysis

The determination of cardiac chamber size and function is of utmost importance for both diagnostic and prognostic considerations in the young with various cardiac abnormalities (18, 19). To date, evaluation of cardiac volume and function by manually corrected 3D echocardiography, without foreshortening the true LV apex, has been validated against radionuclide angiography and CMR in both children and adults (8, 20, 21, 22, 23). Moreover, the American Society of Echocardiography jointly with European Association of Cardiovascular Imaging have currently recommended the use of 3DE quantification of left heart chambers when possible (4). In spite of all the advantages associated with 3DE image display, the 3DE technology with semi-automated algorithm is not so appealing to the busy pediatric and adult cardiologists due to the cumbersome workflow. Thus, the fully automated HeartModel program for 3D cardiac volume determination and LVEF calculation may close the gap between clinical practice and 3D echocardiography.

To date, several recent publications indicated that HeartModel had strong correlations with the expert manual 3DE and CMR (*r* = 0.84–0.97), high reproducibility and shorter analysis time in adult patient group (14, 15). Using the initial version of HeartModel, Tsang and coworkers reported that LVEF was underestimated and automated LVEDV, LVESV and LAV at LVES were overestimated in HeartModel when compared with manual 3DE measurements (14). Still, using the improved version of HeartModel with a default setting of 50% for the global LV boundary for automatic contouring, Spitzer and coworkers demonstrated that only LVEDV were underestimated by HeartModel compared to manual 3DE measurements, with no significant differences in LVESV, LVEF and LAV at LVES (15). Nevertheless, these studies were executed in adults with age ranging from 35 to 86 years, and there were no available data for the application of HeartModel in the adolescent population with or without congenital heart disease. Actually, the automated software adopts a unique adaptive analytics algorithm that derived from extensive trainings using approximately one thousand 3DE datasets of varying image quality in patients with a wide range of function and morphologies (14), which may not encompass adequate echo images from Chinese adolescents. Of note, Kishi and coworkers observed ethnic differences in LV structure and LV function in young adults (24). Thus, it is essential to investigate the feasibility and accuracy of HeartModel in the Chinese adolescent counterparts, whose cardiac chamber size was usually smaller than Western patients. In our adolescent group, we observed an excellent correlation between HeartModel and expert manual 3DE method for estimation of LVEDV, LVESV, LVEF and LAV at LVES (*r* = 0.875–0.965, all *P* < 0.001), using endocardial contouring settings at 40% at end-diastole and 8% at end-systole set by our center. The automated LVEF measurement using HeartModel was accurate and similar to the expert manual measurements, whereas LV volume measurements using this automated program were slightly overestimated. Hence, we can infer that HeartModel represents an accurate and rapid method in pediatric clinic to quantify cardiac volumes and LVEF from 3DE datasets when endocardial boundary recognition is properly setting.

### Automated LAV analysis

Importantly, HeartModel is the first automated technique designed to quantify LAV from 3DE datasets. It has been well established that increased LA volume is an independent marker of adverse cardiovascular outcomes (20, 25, 26), and the latest guidelines have highlighted the importance of LA volume in the context of the evaluation of LV diastolic function (4). In the young adults, we found that the automated LAV values at LVES from the automated 3DE model were slightly larger than the expert manual 3DE measurements, which was in line with the results by Tsang and coworkers (14). Furthermore, the reproducibility of automated LAV measurements was better than previously published inter-observer variability using manual 3DE software (14). This finding is of particular importance, as poor reproducibility would contribute to misclassification of patients with LV diastolic dysfunction (21).

### Reproducibility

In previous semi-automated 3DE studies, the inter-observer variability has ranged from 5% to 15% for LVEDV, 6% to 18% for LVESV, 5% to 21% for LVEF and 5% to 17% for LAV (5, 14, 20, 27, 28, 29, 30, 31, 32). Interestingly, in our study with automated workflow, reproducibility of cardiac volumes and LVEF was excellent and the intra-/inter-observer variability was quite low (<1%), even performed by two observers with different levels of experience (novice and expert). In contrast, the manual 3DE measurements resulted in higher inter-observer and intra-observer variability (3.5–17.4%), which is similar to the previous publications (5, 14, 20, 27, 28, 29, 30, 31, 32), implying that manual 3DE contouring may not be ideal for clinical application (33). Furthermore, the automated technique had excellent test–retest reproducibility. All these findings have presented clinical significance, as observer variability is always an important issue in the echocardiographic laboratories with multiple readers, and the automated workflow with great reproducibility could potentially reduce reader measurement variability and further improve the efficiency in longitudinal assessment during the clinical course. Thus, this automated 3DE program is a significant step forward in promoting wider clinical adoption of 3DE for quantifying cardiac function in a variety of heart diseases.

### Automated program efficiency

Likewise, our study found that the automated 3DE model is significantly faster than expert manual 3DE analysis, approximately a tenth of the time using manual method. Specifically, an operator with minimal experience in 3D volumetric quantification needs only 20–40 s to perform automated cardiac measurements, thus, can help overcome the time-consuming nature of the conventional 3DE analysis that currently limits its use. Moreover, the significant reduction in time has the potential to allow routine use of 3D echo technology in busy echo laboratories.

### Study limitations

The main limitation in the present study is lack of a ‘gold standard’ like CMR to validate the automated technique. However, the prototype-automated software has been validated against CMR in a large group of adult patients at The University of Chicago (14). Second, the study subjects did not include the patients with complex cardiac structural anomaly; thus, future studies in varied types of congenital heart disease are needed. Third, although few patients required endocardial contour correction in this study, it still affects the automated nature of the program. Fourth, patients with poor endocardial visualization were excluded in our study, which results in selection bias. However, the real-world challenge of suboptimal acoustic windows is a physical reality for both 2D and 3D echocardiography. Finally, our study only covered a small number of adolescent subjects in a single-center study, thus a larger study is required to further confirm our results.

## Conclusions

Automated simultaneous quantification of LA and LV volumes and LVEF with HeartModel is feasible, accurate and reproducible in Chinese adolescents. Given the simplicity and efficiency of the automated workflow, this promising program has the potential to enable the integration of 3DE volumetric LV and LA measurements into routine pediatric clinical workflows.

## Declaration of interest

Prof. Alex Pui-Wai Lee received research grant and equipment support from Philips Healthcare.

## Funding

This research project was supported by the Health and Medical Research Fund (Ref No.: 12131351), Food and Health Bureau, Hong Kong SAR Government, Peoples’ Republic of China.
